# Poor treatment responses were related to poor outcomes in pediatric B cell acute lymphoblastic leukemia with KMT2A rearrangements

**DOI:** 10.1186/s12885-022-09804-w

**Published:** 2022-08-06

**Authors:** Jinquan Wen, Min Zhou, Yali Shen, Yueting long, Yuxia Guo, Lin Song, Jianwen Xiao

**Affiliations:** 1 Department of Pediatric Hematology, Caihong Hospital of Xianyang, Xi’an, People’s Republic of China; 2 Department of Hematology, Chengdu Women and Children’s Central Hospital, Chengdu, People’s Republic of China; 3 Department of Hematology, Children’s Hospital of Chongqing Medical University, Yuzhong District, Zhongshan 2nd Road, Chongqing, 400014 People’s Republic of China; 4 Ministry of Education Key Laboratory of Child Development and Disorders, Chongqing, People’s Republic of China; 5 Department of Pediatrics, The Second Affiliated Hospital of Guizhou Medical University, Kaili, People’s Republic of China; 6 National Clinical Research Center for Child Health and Disorders, Chongqing, People’s Republic of China; 7 China International Science and Technology Cooperation Base of Child Development and Critical Disorders, Chongqing, People’s Republic of China; 8 Department of Pharmacy, Children’s Hospital of Chongqing Medical University, Chongqing, People’s Republic of China; 9 Chongqing Key Laboratory of Pediatrics, Chongqing, People’s Republic of China

**Keywords:** B cell acute lymphoblastic leukemia, *KMT2A* rearrangement, Pediatric, Treatment response, Minimal residual disease, Prognosis

## Abstract

**Background:**

The *KMT2A* gene, formerly named the *MLL* gene, is rearranged (*KMT2A*r) in 70–75% of infants, 5–6% of children and 10–15% of adult patients with B cell acute lymphoblastic leukemia (B-ALL). The outcome after chemotherapy of pediatric cases remains poor, and only a few studies have investigated the clinical and laboratory features, treatment response and prognosis in Chinese populations.

**Methods:**

A total of 48 B-ALL children with *KMT2A*r were enrolled in the study, and clinical and laboratory data were collected and analyzed by age group. The relationship between prognosis and traditional risk factors and treatment response was investigated for these patients who received chemotherapy.

**Results:**

The 48 enrolled patients included 28 males and 20 females; 18 (37.50%) or 30 (62.50%) patients were an age of < 12 m (infant B-ALL) or of > 12 m at onset. An initial WBC count of 300 × 10^9^/L was detected in 7 (14.58%) patients; testicular leukemia (TL) or central nervous system involvement was found in 5 (10.41%) or 3 (6.25%) patients, respectively. Statistical differences were not found in the age groups of sex or initial WBC count, whereas TL was more common in the infant group (*P* < 0.05). 11q23 was detected in 18 patients; *KMT2A*r was detected in 46 (95.83%) or 45 (93.75%) patients by FISH or multiplex RT–PCR technology, respectively; RNA-seq data were obtained for 18 patients, and 3 patients with uncommon *KMT2A*r were identified. *KMT2A-AFF1*, *KMT2A-MLLT3* and *KMT2A-MLLT1* were the most common transcripts. Statistical differences were not found in treatment response by age groups, including dexamethasone induction, bone marrow (BM) smear status and minimal residual disease (MRD) level at different time points (TP), treatment-related mortality (TRM), or complete remission (CR) rate (*P* > 0.05); MRD levels monitored by FCM or PCR were unequal at the same TP. Four patients died of treatment, and TRM was 8.33%; 40 patients achieved CR, and the CR rate for the cohort was 83.33%. Seven patients quit, 15 patients relapsed, and the 5 yr cumulative relapse rate was 59.16 ± 9.16%; the 5 yr prospective EFS (pEFS) for patients who were included or excluded from the TRM group was 36.86 ± 8.48% or 40.84 ± 9.16%, respectively. Multivariate analysis for prognosis and hazard ratio was performed for 37 patients without TRM and revealed that an initial WBC count of > 300 × 10^9^/L and a positive level of FCM-MRD were strongly related to a poor outcome for B-ALL patients with *KMT2A*r (*P* < 0.05).

**Supplementary Information:**

The online version contains supplementary material available at 10.1186/s12885-022-09804-w.

## Background

The lysine methyltransferase 2A (*KMT2A*) gene rearrangement, formerly known as the mixed lineage leukemia (*MLL*) gene or *HRX* rearrangement, occurs in 70–75% of infants, 5–6% of elderly children or 10–15% of adult patients with precursor B cell acute lymphoblastic leukemia (B-ALL) [1–3]. More than 90 partner genes for the *KMT2A* gene have been identified and 130 genes transcript had been found, and the *KMT2A-AFF1* (*MLL-AF4*) transcript is the most common subtype. Transcripts of *KMT2A* rearrangements (*KMT2A*r) are considered high-risk fusion genes in precursor B-ALL populations regardless of age, and long-term event-free survival (EFS) rates are less than 40–60%, even with intensive chemotherapy in developed or developing countries [4–6]. Allogeneic hematopoietic stem cell transplantation (AHSCT) provides more than 70–80% of the EFS rate in noninfant pediatric B-ALL patients with *KMT2A*r at first complete remission (CR), but EFS of infant patients or ≥ CR2 patients or not CR patients remains poor [7, 8].

With the development of risk adaptive therapy, the prognosis of *KMT2A*r B-ALL has improved, negative MRD levels at end of remission induction presented with 60.2% of 6 yr EFS of for infants’ group; therefore, it is important to distinguish these patients who did not benefit from chemotherapy at CR1. The study was designed to investigate the outcomes and prognostic factors of B-ALL children with *KMT2A*r.

## Patients and methods

### Patients

Newly diagnosed pediatric B-ALL patients with *KMT2A*r were enrolled in the study. These patients were admitted to the Children’s Hospital of Chongqing Medical University (CHCMU), Chengdu Women and Children Central Hospital (CWCCH), Caihong Hospital of Xianyang (CHX) and the Second Affiliated Hospital of Guizhou Medical University between January 2015 and December 2020. These patients were an age of < 18 yrs at diagnosis, and *KMT2A*r was defined as at least one of t(?; 11q23) detected by chromosomal karyotype and/or fluorescence in situ hybridization (FISH), *KMTA* transcript identified by reverse-transcriptase polymerase chain (RT–PCR) and/or RNA sequencing (RNAseq) [1, 2, 9]. Exclusion criteria included patients with secondary leukemia, Burkitt leukemia (BL), mixed phenotype acute leukemia (MPAL), patients with coexisting specific fusion genes, and patients who had received chemotherapy before admission. Five patients were excluded, and 48 patients were enrolled in the study. The clinical, laboratory and prognostic data were collected and retrospectively analyzed. The study was approved by the Institutional Ethics Committee of CHCMU, and an informed consent form was signed by the guardians of these patients and/or the patients.

Enrolled patients received bone marrow (BM) aspiration and/or BM biopsy at initial diagnosis. The diagnosis of B-ALL was based on FAB classification and flow cytometry (FCM); Chromosomal karyotype was evaluated according to the International System of Human Cytogenetic Nomenclature of 2009 (ISCN-2009); interphase FISH of *KMT2A*r, *ETV6-RUNX1*, *BCR-ABL1*, *MYC* and *PDGFRb* were performed as delineated in a literature report [9, 10]; common fusion genes, included *KMT2A*r transcripts (*KMT2A-AFF1*, *KMT2A-MLLT4*, *KMT2A-MLLT3*, *KMT2A-MLLT10*, *KMT2A-MLLT6*, *KMT2A-EPS15*, *KMT2A-MLLT11*, *KMT2A-FLNA, KMT2A-ELL, KMT2A-MLLT1* and *dupKMT2A*), were screened by multiplex nested RT–PCR (multiplex RT–PCR) [9–11]; Tumor DNA and/or total RNA samples of possible patients were obtained from BM samples at initial diagnosis, whole-exome sequencing (WES) and/or RNA sequencing (RNAseq) were performed by next-generation sequencing (NGS) as found in the literature and as we previously described [9, 10]. The positive KMT2Ar transcript results obtained by multiplex RT–PCR and/or RNA-seq were confirmed by split RT–PCR or quantitative real-time RT–PCR (qRT–PCR).

### Treatment protocol and therapeutic evaluation

The patients were treated with the Chinese Children Cancer Group ALL 2015 protocol (CCCG-ALL-2015), and intrathecal injection (IT) for the prophylaxis of central nervous system leukemia (CNSL) was administered as the protocol required. The criteria for CNSL or testicular leukemia (TL) were defined as described in the literature. The treatment protocol was classified into induction, consolidation, intermediate treatment, reinduction and maintenance treatment [11, 12].

Complete blood counts (CBCs) were detected after four days of dexamethasone induction, and absolute blast cell counts were recorded. Blast cell counts < 1 × 10^9^/L or ≥ 1 × 10^9^/L were defined as good dexamethasone response (DGP) or poor dexamethasone response (DPR), respectively. BM studies were conducted at the following different time points (TPs): Day 19 of induction remission (TP1) and Day 46 of induction remission (TP2) or prior to consolidation (TP3). The remission status of BM smear samples was recorded as follows: M1 (CR, leukemic cells < 5%), M2 (leukemic cells 5–25%) and M3 (leukemic cells ≥ 25%). Minimal residual disease (MRD) level monitoring was performed by FCM and RT–PCR techniques; 10^–4^ was considered the limitation of FCM-MRD monitoring, and negative values were regarded as the cutoff values of PCR-MRD monitoring [9, 11].

These *KMT2A*r B-ALL patients were considered the intermediate risk (IR) group, and the high risk (HR) group was classified as patients with ages of < 6 m and WBC counts of ≥ 300 × 10^9^/L at initial diagnosis or an FCM-MRD level monitoring of ≥ 1% at TP2. Details of the protocol and the risk group classification are listed in the Supplementary Materials.

### Statistical analysis

Complete remission (CR), BM relapse or extramedullary relapse were defined as diagnostic criteria, and EFS was defined as the time from diagnosis to the date of last contact for event-free survivors or to the first adverse event (not CR status after induction remission, relapse or refractory disease, death from any cause, secondary cancer or loss to follow-up). Following 31 January 2022, data on the clinical and laboratory findings, treatment responses, treatment-related mortality (TRM) and EFS of the enrolled patients were collected and retrospectively analyzed. *SPSS 20.0* software (SPSS Inc., Chicago, IL) or *GraphPad Prism 8.02* was used for statistical analysis and/or chart drawing. The impact of clinical and laboratory findings on EFS was assessed with the *Kaplan–Meier* method, and comparisons were made with the *log-rank* test. Multivariate analysis for prognosis and hazard ratio were performed using the *Cox* regression model. All probabilities reported were two-tailed, and *P* values of < 0.05 were regarded as significant differences.

## Results

### Baseline data

**Fig. 1:**
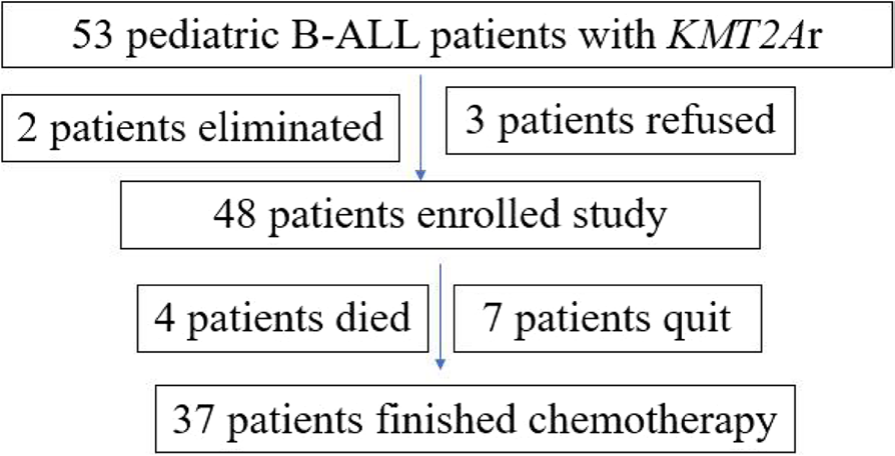
Patients enrolled the study

A total of 886 newly diagnosed B-ALL patients were enrolled the period, which included 857 non-infant patients (≥ 12 m) and 29 infant patients (< 12 m). 53 B-ALL patients with *KMT2A*r were admitted in the period, 2 non-infant patients were eliminated owing to errors in immunophenotyping (1 patients was BAL, 1 patient diagnosed MPAL) *KMT2A*r B-ALL were found in 65.51% (19 of 29) infant patients and in 3.73% (32/857) non-infant patients respectively. Chemotherapy was refused for 3 patients (1 infant patients and 2 non-infant patient), 48 consecutive patients were treated with the CCCG-ALL-2015 protocol and were enrolled in the study (Fig 1).

The clinical and laboratory findings of the 48 enrolled patients are listed in Table 1. The study group included 28 males and 20 females (1.53:1), and cancer family histories were found in 3 (6.25%) patients. The range, median or average values of age, white blood cell (WBC) count and lactate dehydrogenase (LDH) level at diagnosis were 3–173 m (18.50 m, 45.19 ± 7.41 m), 1.94–652.28 × 10^9^/L (98.00, 149.79 ± 23.08 × 10^9^/L) and 201–4059 U/L (874.50, 1005.90 ± 113.447 U/L), respectively. Initial WBC counts ≥ 50 × 10^9^/L or 300 × 10^9^/L were detected in 34 (70.83%) or 7 (14.58%) patients, respectively; LDH levels ≥ twofold of normal values (upper limit value: 330 U/L) were detected in 31 (64.58%) patients. Testicular or CNS involvement was found in 5 (10.41%) or 3 (6.25%) patients in the cohort.

**Table 1:** Characteristics of the enrolled 46 patients

Characteristics	Infant group (*n* = 18)	Non infant group (*n* = 30)	Fisher or t test	*P* =
Sex				
Male	11	17		> 0.9999
Female	7	13		
WBC count	153.49 ± 37.37	147.57 ± 29.82	t = 0.1239, df = 36.80	0.9021
≥ 50 × 10^9^/L	14	20		0.5206
< 50 × 10^9^/L	4	10		
LDH (U/L)	1114.13 ± 202.13	940.96 ± 136.44	t = 0.7101, df = 32.11	0.4828
≥ 2 N	13	18		0.5358
< 2 N	5	12		
Extramedullary involvement				
Negative	12	28		0.0396
Positive (CNSL, TL)	6 (2,4)	2 (1,1)		
KMT2Ar distribution				
KMT2A-AFF1	10	14		0.7661
Non KMT2A-AFF1	8	16		

Eighteen (37.50%) patients were < 12 m (infant B-ALL) at onset, and significant differences were not found in the age groups of sex or WBC count. The LDH level (*P* > 0.05) at initial diagnosis and extramedullary involvement (TL) were more common in the infant group (*P* < 0.05). The baseline data of the infant and noninfant groups in the cohort were similar (Table 1).

Chromosomal karyotypes were evaluated for 48 patients, and 8 patients were not evaluated. Normal karyotypes (46,XX/46,XY) were detected in 20 patients, and abnormal karyotypes were found in 20 patients. t(?;11q23) was detected in 18 patients, the detection rate of t(?;11q23) in the 40 analyzed patients was 45.00%. A DNA index of < 1.15 was not found in these 40 analytical patients. *KMT2A*r was detectable in 46 (95.83%) or 45 (93.75%) patients by FISH or multiplex RT–PCR, respectively. RNA-seq data were obtained for 18 of the 48 patients. The same *KMT2A*r transcript was obtained in 15 patients. Three patients with uncommon *KMT2A*r transcripts were determined by RNA-seq. Two of the patients (*KMT2A-CT45A1*, *KMT2A-CLTC*) were not detected by multiplex RT–PCR, and 1 patient (*KMT2A-USP2*) was undetectable by both FISH and multiplex RT–PCR.

All 48 detectable cases of *KMT2A*r transcript results were confirmed by split RT–PCR, which included 24 (50.00%) patients with *KMT2A-AFF1* transcripts, 13 (27.08%) patients with *KMT2A-MLLT3 transcripts*, 5 (10.42%) patients with *KMT2A-MLLT1* transcripts, and 1 (2.08%) patient with *KMT2A-EPS15*, *KMT2A-MLLT10*, *KMT2A-FLNA*, *KMT2A-CLTC*, *KMT2A-CT45A1* or *KMT2A-USP2* transcripts (Table 1).

### Treatment response evaluation

Eighteen patients were classified as the infant group, blast cell counts at Day 5 of dexamethasone induction were evaluated, and negative or DGP were found in 6 or 11 cases, respectively. One patient died of sepsis before TP1, BM smears demonstrated 16 patients with M1 and 1 patient with M3; FCM-MRD level presented with < 0.01% or ≥ 0.01% in 6 or 11 patients, respectively, FCM-MRD level presented with < 1% or ≥ 1% was found in 12 or 5 patients, respectively; PCR-MRD level was determined, 6 patients were negative and 11 patients were positive. However, the results of FCM-MRD and PCR-MRD levels were not synchronized. Two patients died of sepsis and invasive fungal infections. BM samples were obtained from 15 patients at TP2, which showed that 12 patients were defined as CR by BM smear, and the CR rate was 66.66% in the total infant group. MRD levels were negative (< 0.01%) in 6 patients monitored by the FCM-MRD technique, and negative results were found in 7 patients by the PCR-MRD technique.

Thirty patients were defined as the noninfant group, and negative results or DGP were found in 14 or 18 patients, respectively. At TP1, BM smears demonstrated 28 patients with M1 and 2 patients with M2 or M3; FCM-MRD level presented with < 0.01% or ≥ 0.01% in 18 or 12 patients, respectively, FCM-MRD level presented with < 1% or ≥ 1% was found in 19 or 11 patients, respectively; negative or positive MRD results were found in 10 or 20 patients by PCR technique, FCM-MRD and PCR-MRD level were not synchronized. One patient died of sepsis, and BM samples were obtained from 29 patients at TP2. These 28 patients were regarded as CR by BM smear, and the CR rate was 93.33%. MRD levels were negative in 21 or 17 patients monitored by FCM or PCR, respectively.

Statistical differences were not found in the treatment response by age group, including dexamethasone induction, BM smear status and MRD level at different TPs, CR rates or TRMs (*P* > 0.05). The data are listed in Table 2. MRD levels monitored by FCM or PCR were unequal at the same TP, revealing that both FCM-MRD and PCR-MRD should be monitored for *KMT2A*r B-All populations for treatment response evaluation.

**Table 2:** Treatment response by age groups of the 48 enrolled patients

Treat respond	Infant group (*n* = 18)	Non infant group (*n* = 30)	Fisher test	*P* =
dexamethasone induction (*n* = 48)				
Negative	6	14		0.5461
Positive	12	16		
DGR	11	18		> 0.9999
DPR	7	12		
BM status at TP1^a^(*n* = 47)				
M1	16	28		> 0.9999
M2 or M3	1	2		
FCM-MRD level at TP1^a^ (*n* = 47)				
< 0.01%	6	18		0.1351
≥ 0.01%	11	12		
< 1%	12	19		0.7526
≥ 1%	5	11		
PCR-MRD level at TP1^a^ (*n* = 47)				
Negative	6	10		> 0.9999
Positive	11	20		
BM status at TP2^a^(*n* = 45)				
CR	12	28		0.1073
Not CR	3	1		
FCM-MRD level at TP2^a^ (*n* = 45)				
< 0.01%	6	21		0.0526
≥ 0.01%	9	8		
PCR-MRD level at TP2^a^ (*n* = 45)				
Negative	7	17		0.5213
Positive	8	11		
Treat related mortality (*n* = 48)				
Yes	3	1		0.1415
no	15	29		

^a^ for survival patients

### Analysis of prognosis and related risk factors

Of the 44 patients without TRM, 40 patients obtained CR status, and 4 patients did not achieve CR evaluated by BM smear. Seven (5 male and 2 female) of these 40 CR patients left the study because of family choice. Details regarding the patients that left the study are listed in Table 3. Chemotherapy was continued for 33 patients. After 31 January 2022, no further patients died of treatment, and 15 patients (BM relapsed: 14 patients; combination relapsed of BM and CNS: 1 patient) relapsed.

**Table 3:** Basic data of these quit patients

Patient	gender	Age (m)	WBC count (× 109/L)	KMT2Ar subtype	Extramedullary involvement	Blast cell at Day 5 (× 109/L)	D19			D46		
							BM smear	FCM-MRD (%)	PCR-MRD	BM smear	FCM-MRD (%)	PCR-MRD
1	M	53	277.97	KMT2A-AFF1	N	3.776	CR	6.21%	Negative	CR	3.54%	Positive
2	M	4	652.28	KMT2A-MLLT1	TL	69.0477	CR	0.50%	Positive	CR	0.00%	Positive
3	M	145	595	KMT2A-AFF1	N	0.8073	CR	1.73%	Positive	CR	0.84%	Positive
4	F	7	136.08	KMT2A-AFF1	N	0	CR	0.00%	Negative	CR	0.00%	Negative
5	M	173	191.89	KMT2A-AFF1	TL	0	CR	0.04%	Positive	CR	0.00%	Positive
6	F	26	23.71	KMT2-MLLT3	N	1.0504	CR	9.05%	Positive	CR	0.11%	Positive
7	M	12	219.52	KMT2A-AFF1	N	36.7445	CR	0.52%	Positive	CR	0.44%	Positive

**Fig. 2:**
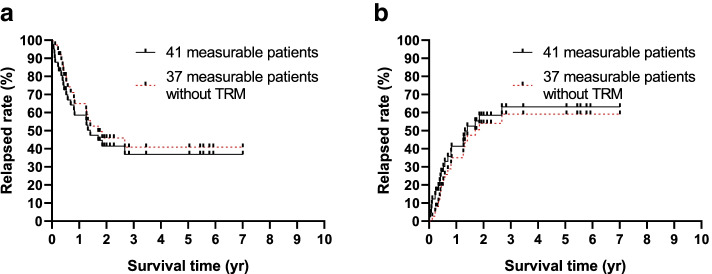
Survival of the cohort

In general, 48 patients were enrolled the study, 4 patients died of treatment and TRM was 8.33%; 40 patients got CR and CR rate for the total 48 patients was 83.33%. 7 patients quit the study. Median follow up for the 37 patients without TRM was 15.48 m, 15 patients relapsed and 5 yr cumulative relapse rate for the 37 patients was 59.16 ± 9.16%; 5 yr prospective EFS (pEFS) for patients included TRM (41 patients) or excluded TRM (37 patients) was 36.86 ± 8.48% or 40.84 ± 9.16% respectively (Fig 2).

Two of the 4 patients without CR underwent reinduction by hyper CVAD-based chemotherapy and achieved CR evaluated by BM smear. MRD levels were monitored by both FCM and PCR techniques. One patient achieved negative results for both FCM and PCR-MRD levels, and 1 patient presented with negative FCM-MRD levels and positive PCR-MRD levels. Five of 14 relapsed patients received reinduction, and CR status was achieved in 1 patient. CD19 CAR-T therapy was performed for 2 of the 4 patients, and both patients achieved CR evaluated by BM smear, FCM and PCR-MRD.

**Fig. 3:**
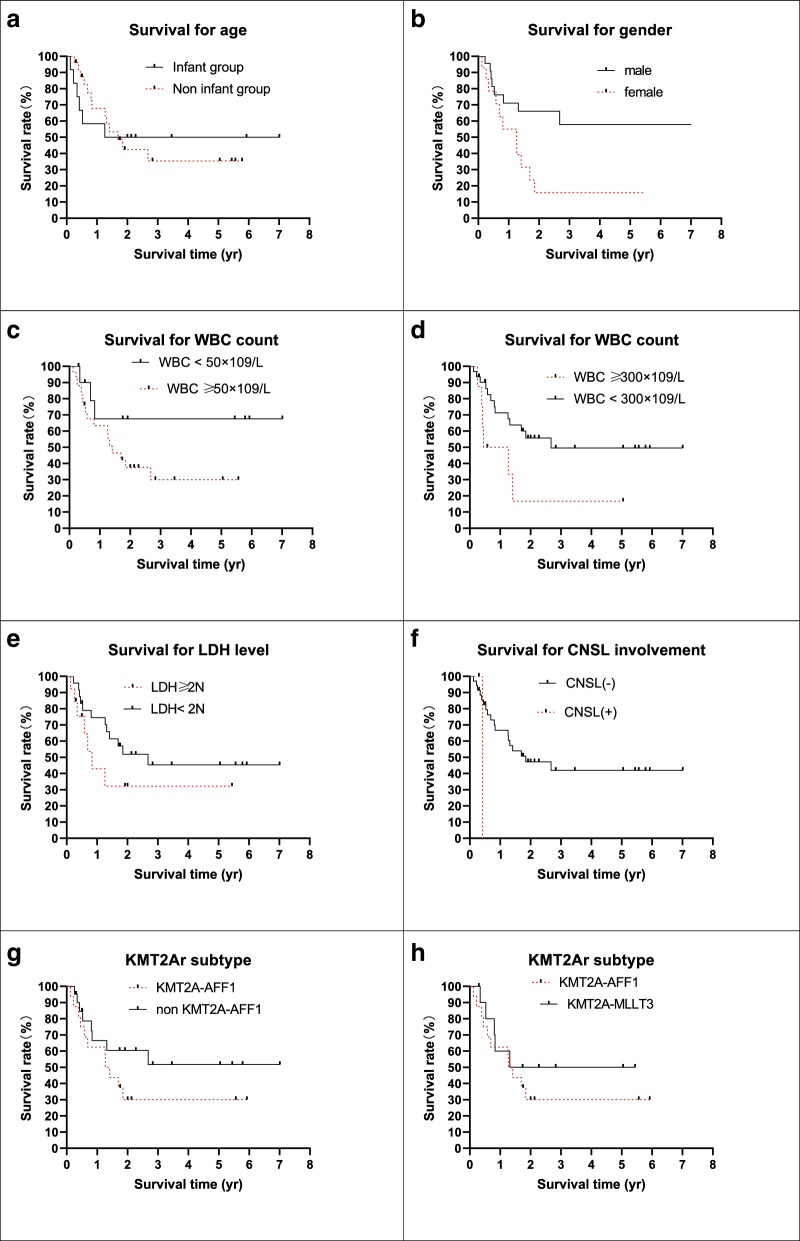
Survival of the measurable patients by traditional risk factors

Hazard factors for the study were collected and analyzed for the 37 measurable patients without TRM. Traditional risk factors are listed in Table 4 and Fig. 3. The 5-year pEFS was longer in the male group, with WBC counts ≥ 50 × 10^9^/L and 300 × 10^9^/L (*P* < 0.05). Significant differences were not found in the different age and initial LDH level groups (*P* > 0.05). The CNSL data were too small to be compared. Subtypes of *KMT2A*r were also evaluated, and the prognosis of the *KMT2A-AFF1* transcript was lower than that of the other subtypes, but the difference was not statistically significant (*P* > 0.05).

**Table 4:** Traditional risk factors and outcomes

Demographics	*n* = 37^a^	%	pEFS	*P* =
Age group				
Infant group	12		50.00 ± 14.43%	0.9974
Noninfant group	25		35.35 ± 11.28%	
Gender				
Male	23		57.80 ± 12.00%	0.0160
Female	14		15.71 ± 10.18%	
WBC count				
≥ 50 × 10^9^/L	25		30.03 ± 10.44%	0.1178
< 50 × 10^9^/L	12		67.50 ± 15.51%	
≥ 300 × 10^9^/L	6		16.67 ± 14.83%	0.0342
< 300 × 10^9^/L	31		49.57 ± 10.38%	
LDH (U/L)				
≥ 2 N	24		32.23 ± 15.06%	0.1762
< 2 N	13		45.36 ± 11.07%	
CNSL involvement				
CNSL-	35		41.95 ± 9.34%	-
CNSL +	2		-	
KMT2Ar subtype				
KMT2A-AFF1	16		30.00 ± 11.78%	0.1745
Non KMT2A-AFF1	21		51.83 ± 12.88%	
KMT2A-AFF1	16		30.00 ± 11.78%	0.4154
KMT2A-MLLT3	11		50.00 ± 15.81%	

^a^ patients without TRM

**Table 5:** Treatment response and outcomes

Demographics	*n* = 37^a^	%	pEFS	*P* =
Dexamethasone response				
Negative	17		65.00 ± 12.68%	0.0442
Positive	20		23.44 ± 10.61%	
DGR	23		60.71 ± 10.87%	0.0269
DPR	14		15.71 ± 10.18%	
BM status at TP1				
M1	34		43.09 ± 9.52%	-
M2 or M3	3		-	
FCM-MRD level at TP1				
< 0.01%	22	%	55.55 ± 11.24%	0.0221
≥ 0.01%	15	%	22.86 ± 12.90%	
< 1%	24	%	52.38 ± 10.70%	0.0175
≥ 1%	13	%	15.39 ± 13.01%	
PCR-MRD level at TP1				
Negative	13	%	76.92 ± 11.69%	0.0031
Positive	24	%	19.13 ± 9.43%	
FCM-MRD level at TP2				
< 0.01%	25	%	57.69 ± 12.01%	< 0.0001
≥ 0.01%	12	%	8.33 ± 7.98%	
PCR-MRD level at TP2				
Negative	23	%	64.17 ± 10.93%	< 0.0001
Positive	14	%	8.33 ± 7.93%	

^a^ patients without TRM

**Fig. 4:**
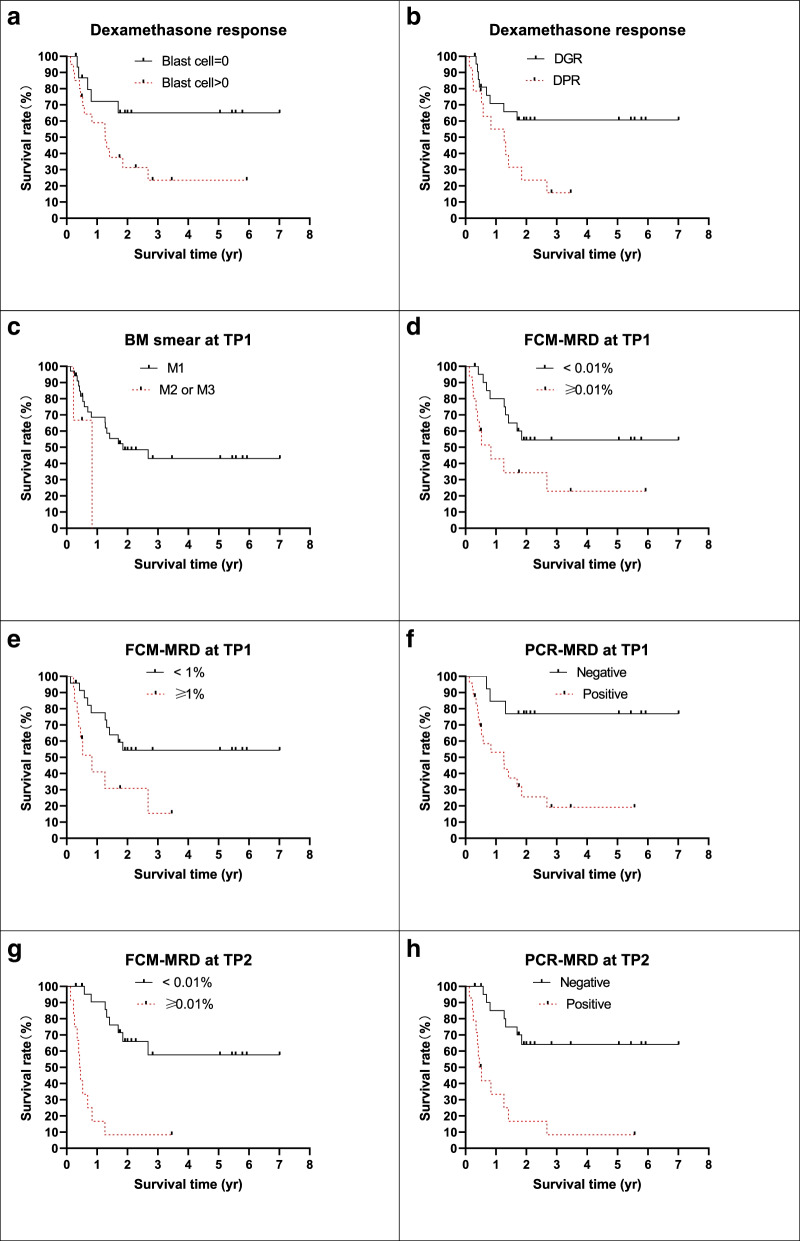
Survival of the measurable patients by treatment respond

The relationship between treatment response and prognosis was also analyzed, and good treatment responses, including dexamethasone induction, BM smear at TP1, FCM-MRD or PCR-MRD level at TP1 and TP2, were related to better prognosis (Table 5, Fig 4). It should be noted that positive FCM-MRD results or positive PCR-MRD results were both strongly related to worse outcomes, and 5-year pEFS was < 10% when patients presented with positive MRD levels at TP2.

Multivariate analysis for prognosis and hazard ratio was performed using the *Cox* regression model (Table 6), which revealed that the female sex, an initial WBC count of > 300 × 10^9^/L and a positive level of FCM-MRD were related to poor outcome for B-ALL patients with *KMT2A*r; however, 5 males and 2 females achieved CR but quit chemotherapy, and larger samples and multicenter studies are needed to confirm this hypothesis. It seems that an initial WBC count > 300 × 10^9^/L and FCM-MRD level were the key risk factors in pediatric B-ALL patients with *KMT2A*r.

**Table 6:** Multivariate analysis of prognoses and hazard ratios by the *Cox* regression model

Multivariate analysis of prognoses and hazard ratios by the *Cox* regression model								
		B	SE	Wald	df	Sig	Exp(B)	95.0% *CI*
Gender	Male vs. female	1.218	0.500	5.931	1	0.015	3.380	1.268–9.008
Age	Infant vs. non infant	1.256	NA	NA	1	0.262	NA	NA
WBC count (× 10^9^/L)	> 300 vs. < 300	-1.465	0.592	6.136	1	0.013	0.231	0.072–0.736
Dexamethasone induction	DGR vs. DPR	1.203	NA	NA	1	0.314	NA	NA
TP1 FCM-MRD	Pos. vs. Neg	0.080	NA	NA	1	0.778	NA	NA
TP1 PCR-MRD	Pos. vs. Neg	1.254	NA	NA	1	0.263	NA	NA
TP2 FCM-MRD	Pos. vs. Neg	-1.465	0.592	6.136	1	0.013	0.231	0.072–0.328
TP2 PCR-MRD	Pos. vs. Neg	1.847	NA	NA	1	0.174	NA	NA

## Discussion

The *KMT2A* gene is located at 11q23.3 and coexists with its partner genes. *KMT2A*r encodes its relevant *KMT2A* protein and acts as an epigenetic regulator of transcriptional initiation through histone-3 lysine-4 methylation of target gene promoter regions, regulators of hematopoietic cell proliferation and differentiation and the homeobox A gene cluster. Deregulation of these genes initiates the development of leukemia through inhibition of proper hematopoietic development [13, 14]. The *KMT2A*r fusion genes with their breakpoints code for their related proteins and are regarded as poor factors for both B-ALL and acute myeloid leukemia (AML) [13–15].

Pediatric or adult B-ALL with t(v;11q23.3) was regarded as a specific entity of recurrent genetic abnormalities in B-ALL by the WHO-2016 Classification of Hematopoietic and Lymphoid Tissues Tumors [14, 15]. *KMT2Ar* accounts for 70% of infant ALL cases, whereas it is not common in children older than 1 year [1, 2]. With different partner genes, more than 130 fusion gene transcripts have been identified; t(4;11)(q21;q23) and its transcript *KMT2A-AFF1* (formerly named *MLL-AF4* fusion gene) and t(9)(11)(p22;q23) and its transcript *KMT2-MLLT3* (*MLL-AF9*) remain the most common subtype in both infant and elderly pediatric patients [1, 8, 16]. Our data also presented with these findings. Uncommon *KMT2A*r transcripts were determined in 3 patients in the cohort by RNAseq, and we believe that *KMT2A-CT45A1, KMT2A-CLTC*it and *KMT2A-USP2* transcripts were the 1^st^ reported in the Chinese population. It also revealed that the RNA-seq technique was more sensitive than the FISH or the PCR technique for transfusion detection, i.e., rare *KMT2A*r transcripts.

In our study, 4 patients died of severe infection, and the TRM was 8.33%. The data were higher than those of developed countries, partially due to the socioeconomic conditions in developing countries [6, 8–10, 14]. A total of 4 patients, including 3 of 18 infants or 1 of 30 elderly patients, died of TRM in the course of induction remission, which indicated that intensive care, antimicrobial prophylaxis and positive pressure rooms are important for neutropenic conditions.

The baseline data of the infant and noninfant groups were similar except that TL was more common in the infant group, and multiple centers and large data are needed to identify TL. The treatment response of the enrolled patients was evaluated and classified by age group. Statistical differences were not found between infant and noninfant patients, including dexamethasone induction response, BM smear, and MRD level determined by FCM or PCR at different TPs. A literature review showed that infant patients achieved a better treatment response (FCM-MRD level) when treated by the Interfant-06 protocol [17].

*KMT2A*r B-ALL has a poor treatment response and prognosis. The CR rate in our cohort was 83.33%, and the 5-year pEFS for patients who did not have TRM was 40.84 ± 9.16%. These data were far lower than those of patients with B-ALL with good indicators, *ETV6-RUNX1* transcripts or hyperploid karyotypes (> 95%, > 80–90%) [6, 7, 10].

The literature showed that an initial WBC count > 300 × 10^9^/L was related to poor outcome [17], and our data also supports this hypothesis. EFS was longer in males than in females in the cohort, but the sample size was too small to confirm it. *KMT2Ar* is a reverse prognostic factor for B-ALL regardless of different *KMT2A*r subtypes in our study. The result was different from those in literature reports, and we think it was partly due to different treatment strategies [1, 18, 19]. Further studies are needed to confirm this.

Treatment response was a strong indicator for *KMT2A*r B-ALL patients. Patients who were sensitive to dexamethasone induction presented with prolonged 5 yr pEFS compared with these patients who were not. Similar results were identified in early studies for prednisone induction [6, 10], revealing that the treatment response to glucocorticoids remains a helpful method for predicting the prognosis of pediatric B-ALL with *KMT2A*r.

In our study, pEFS for patients with positive MRD level at TP1 for FCM (cutoff value < 0.01%) or RT-PCR were < 20 -30%; whereas pEFS for patients with positive MRD level at TP2 were < 10–15%. Multivariable analysis for prognosis and hazard ratio was performed, it reveals that initial WBC count > 300 × 10^9^/L or positive FCM-MRD level at TP2 was related to poor outcome. MRD level monitored by FCM and/ or RT-PCR were good predictor of outcome for pediatric B-ALL patients at any time points [17, 18], sensitivity and specificity for MRD monitored by FCM or RT-PCR at different TP remained different, further studied were need for the values for prognosis prediction in these patients. Infant patients treated with Interfant-06 protocol and presented with negative MRD level obtained with 6 yr EFS of 68.2% (95% CI, 55.2 to 78.1) [17]; 2 patients without CR in our study underwent re-induction by hyper CVAD based chemotherapy and achieved CR. These data prompts that Interfant-06 protocol might be more suitable for infant B-ALL patients with *KMT2A*r [9, 19], we hypothesis that outcome of *KMT2Ar* positive B-ALL patients might be improved by protocol contain high dosage of cytarabine, further experimental and clinical studies were need. Our data reveal that prognosis of patients with poor treatment respond or initial WBC count > 300 × 10^9^/L remained poor, literatures reports showed that B-ALL patients with *KMT2A*r may benefit from allo-HSCT [7], these patients should receive allo-HSCT at CR1.

In conclusion, we assessed the outcomes and risk factors for Chinese pediatric B-ALL patients with *KMT2A*r, outcomes remained poor by conventional chemotherapy. Allo-HSCT at CR1 for patients with risk factors might improve its prognosis; effects of high dose cytarabine had been identified for infant patients, further studies, included high dose novel agents, target agents, were needed to improve outcomes for children and adult patients.

## Supplementary Information

**Additional file 1.**

## Data Availability

The datasets used and/or analysed during the current study were available from the corresponding author on reasonable request.
