# Supplementary material for: Poor treatment responses were related to poor outcomes in pediatric B cell acute lymphoblastic leukemia with KMT2A rearrangements
Source: BMC Cancer. 2022 Aug 6;22:859. doi: 10.1186/s12885-022-09804-w (PMC9357304; doi:10.1186/s12885-022-09804-w)
Supplement: Supplementary file 1 — Additional file 1. [file 12885_2022_9804_MOESM1_ESM.docx]

**Supplementary material**

**TABLES AND FIGURES WITH LEGENDS**

**S-1 CCCG-ALL-2015 protocol, IR/HR group**

| Phase | Drug | Dosage | Duration |
| --- | --- | --- | --- |
| Remission induction | Dex | 8 mg/m^2^/d, po/iv | Days 1-4 |
|  | Pred | 60 mg/m^2^/d, po | Days 5-28, tapering after 9 days |
|  | DNR | 25 mg/m^2^/d, iv | Days 5 and 12 |
|  | VCR | 1.5 mg/m^2^/d (max: 2 mg) iv | Days 5, 12, 19, and 22 |
|  | PEG-asp | 2000 U/m^2^/d, im | Days 6 and 26 |
|  | CTX | 1 g//m^2^/d, iv | Days 29 |
|  | AraC | 50 mg/m^2^, q12h, IH | Days 29 to 35 |
|  | 6-MP | 50 mg/m^2^/d, po | Days 29 to 35 |
|  | TiT | Related to age | Days 5, 8,12, 15, and 19 |
| Consolidation | MTX | 5 g/m^2^/d, iv | Days 1, 15, 29, and 43 |
|  | CF | 15 mg/m^2^, im, q6h, for rescue | 42 h after MTX administration, at least 3 doses |
|  | 6-MP | 25 mg/m^2^/d, po | Days 1 to 56 |
|  | TiT | Related to age | Days 5, 8,12, 15, and 19 |
| Interphase x 5 cycles: every 3 weeks | Dex | 12 mg/m^2^/d, po | Days 1-5 |
|  | DNR | 25 mg/m^2^/d, iv | Day 1 |
|  | VCR | 1.5 mg/m^2^/d (max: 2 mg) iv | Day 1 |
|  | PEG-asp | 2000 U/m^2^/d, im | Day 3 |
|  | 6-MP | 25 mg/m^2^/d, po | Days 1 to 21 |
|  | TiT | Related to age | Day 1 |
| Reinduction of remission | Dex | 8 mg/m^2^/d, po | Days 1 to 7 and 15 to 21 |
|  | VCR |  | Days 1, 8, and 15 |
|  | PEG-asp | 2000 U/m^2^/d, im | Day 3 |
|  | AraC | 2 g/m^2^, q12h | Days 1 and 2 |
|  | TiT | Related to age | Day 1 |
| Maintenance |  |  |  |
| Course-1 (5 cycles): every 4 weeks | MTX | 25 mg/m^2^/d, po | Day 1 and 8 |
|  | 6-MP | 50 mg/m^2^/d, po | Days 1 to 14 |
|  | CTX | 300 mg/m^2^/d, iv | Day 15 |
|  | VCR | 1.5 mg/m^2^/d (max: 2 mg) iv | Day 15 |
|  | AraC | 300 mg/m^2^/d, iv | Day 15 |
|  | DEX | 8 mg/m^2^/d, po | Days 15 to 21 |
|  | TiT | Related to age | Day 15 |
| Course-2 (7 cycles): every 8 weeks | MTX | 25 mg/m^2^/d, po | Days 1, 8, 15, 22, 29, 36, and 42 |
|  | 6-MP | 50 mg/m^2^/d, po | Days 1 to 49 |
|  | CTX | 300 mg/m^2^/d, iv | Day 50 |
|  | VCR | 1.5 mg/m^2^/d (max: 2 mg) iv | Day 50 |
|  | AraC | 300 mg/m^2^/d, iv | Day 50 |
|  | DEX | 8 mg/m^2^/d, po | Days 50 to 56 |
|  | TiT | Related to age | Day 50 |
| Course-3 (2 cycles) | MTX | 25 mg/m^2^/d, po | Days 1 to 56 |
|  | 6-MP | 50 mg/m^2^/d, po | Days 1 to 56 |
| CCCG: China Children Cancer group; ALL acute lymphoblastic leukemia; IR: intermediate risk; HR: high risk | | | |

**S-2 iT for the prophylaxis of CNSL**

| Age (m) | MTX (mg) | AraC (mg) | Dex (mg) |
| --- | --- | --- | --- |
| <12 | 6 | 15 | 2.5 |
| 12-36 | 9 | 25 | 2.5 |
| >36 | 12.5 | 35 | 5 |
| iT: intrathecal injection; CNSL: central nervous system leukemia | | | |

**S-3 Risk group classification**

| IR risk group: all the B-ALL patients with *KMT2A*r. |
| --- |
| HR risk group:   1. Any patients MRD level monitored by flow cytometry ≥ 1% at day 46 of induction remission; 2. B-ALL patients with *KMT2A*r <6m and WBC count ≥ 300×10^9^/L at diagnosis. |
| MRD: minimal residual disease; WBC: white blood cell |
